# An Improved Binary Vector and *Escherichia coli* Strain for *Agrobacterium tumefaciens*-Mediated Plant Transformation

**DOI:** 10.1534/g3.116.029405

**Published:** 2016-05-17

**Authors:** Michael R. Watson, Yu-fei Lin, Elizabeth Hollwey, Rachel E. Dodds, Peter Meyer, Kenneth J. McDowall

**Affiliations:** * Centre for Plant Sciences and School of Biology, Faculty of Biological Sciences, University of Leeds, LS2 9JT, United Kingdom; † Astbury Centre for Structural Molecular Biology, University of Leeds, LS2 9JT, United Kingdom; ‡ School of Molecular and Cellular Biology, Faculty of Biological Sciences, University of Leeds, LS2 9JT, United Kingdom

**Keywords:** *Agrobacterium*, plant transformation, pGreen, *E. coli*, growth defects

## Abstract

The plasmid vector pGreenII is widely used to produce plant transformants via a process that involves propagation in *Escherichia coli*. However, we show here that pGreenII-based constructs can be unstable in *E. coli* as a consequence of them hampering cell division and promoting cell death. In addition, we describe a new version of pGreenII that does not cause these effects, thereby removing the selective pressure for mutation, and a new strain of *E. coli* that better tolerates existing pGreenII-based constructs without reducing plasmid yield. The adoption of the new derivative of pGreenII and the *E. coli* strain, which we have named pViridis and MW906, respectively, should help to ensure the integrity of genes destined for study in plants while they are propagated and manipulated in *E. coli*. The mechanism by which pGreenII perturbs *E. coli* growth appears to be dysregulation within the ColE1 origin of replication.

Central to the study and engineering of plants is their transformation, which is achieved most commonly using *Agrobacterium tumefaciens*, the causal agent of crown galls (or tumors) in dicotyledonous plants (Smith and Townsend 1907). The induction of crown galls is induced by the transfer of T-DNA (Thomashow *et al.* 1980), a segment of a tumor-inducing plasmid (Van Larebeke *et al.* 1974) that is resident in *A. tumefaciens*, into the nucleus of infected plant cells, wherein it is stably integrated into the genome and expressed (Chilton *et al.* 1977). The genes required for the transfer of T-DNA have been identified (Zambryski *et al.* 1989) and are well characterized (Pitzschke and Hirt 2010). Moreover, the segment between the borders of T-DNA can be engineered to contain heterologous DNA while maintaining the ability to be transferred efficiently into plant cells (Joos *et al.* 1983). Indeed, *A. tumefaciens* is used routinely to transform many plant species of academic, agronomical, and horticultural importance (Hooykaas and Schilperoort 1992). To our knowledge, all of the systems that have been developed for this purpose incorporated binary vectors (Hoekema *et al.* 1983; Hellens *et al.* 2000b) that allow propagation in *Escherichia coli*, wherein DNA can be readily cloned and manipulated between the borders of the T-DNA, prior to transfer into *A. tumefaciens* and finally plants (Bevan 1984).

One of the most widely used binary vectors in the *Agrobacterium*-mediated transformation of plants is pGreenII (Hellens *et al.* 2000a). The origin that facilitates pGreenII replication in *E. coli* is derived from plasmid ColE1 (Hershfie *et al.* 1974). An earlier version of pGreenII was reported to be unstable, whereby it acquired DNA from the genome of *E. coli* prior to transfer of plasmid into *A. tumefaciens* (Hellens and Mullineaux 2000). Consequently, the region between the ColE1 origin and the stop codon of the adjacent *npt1* gene, which is convergent and confers resistance to kanamycin, was replaced with the corresponding sequence from pBluescript^©^ (Hellens and Mullineaux 2000), a commercially available and widely used cloning vector that has no reported issues with instability (Alting-Mees and Short 1989).

The pGreen system remains one of the most widely used for plant transformation, featuring annually in around 200 scientific publications over the past 5 yr (as revealed using Google Scholar). While alternative binary vector systems have been developed, *e.g.*, pCambia (http://www.cambia.org/; Hajdukiewicz *et al.* 1994), pGreenII-based constructs can be introduced into plants more efficiently depending on the species or variety, or the genetic marker being selected (Binka *et al.* 2012). Recently a CRISPR/Cas9 toolkit for multiplex genome editing in plants was based on the pGreen, as well as the pCambia, backbone (Xing *et al.* 2014).

As part of a study of *Arabidopsis thaliana MET1*, which encodes a cytosine-DNA-methyltransferase involved in epigenetic gene regulation (Watson *et al.* 2014; Zubko *et al.* 2012), we observed that *E. coli* cells containing a pGreenII-based construct that carries *MET1* produced unusually small colonies on agar plates and were extremely difficult to passage (*i.e.*, subculture). Moreover, this poor growth provided sufficient selective pressure for mutants, some of which had rearrangements of the plasmid, to dominate the population when dense cultures were eventually obtained. The finding that a plasmid-based construct can affect the growth of *E. coli* was not in itself unusual (Betenbaugh *et al.* 1989; Diaz Ricci and Hernandez 2000). However, closer investigation revealed that the pGreenII vector itself without any insert affects the growth of *E. coli* substantially, which in turn places pGreenII-based constructs under considerable selective pressure. As the maintenance of the integrity of cloned DNA is of paramount importance, we have produced a new version of pGreenII that does not affect the growth of *E. coli*. We also describe the selection of a new strain of *E. coli* that better tolerates existing pGreenII-based constructs without reducing plasmid yield.

## Materials and Methods

### Plasmids: sources, propagation, and analysis

The plasmid pGreenII (version 0179; http://www.pgreen.ac.uk/) was obtained from the John Innes Centre (Norwich Research Park, UK). The pMET1-03 plasmid contains the cDNA of *MET1* under the control of the 35S promoter. The *MET1* cDNA sequence was obtained as a 4865 bp *Eco*RI fragment from a previously described construct (Zubko *et al.* 2012) based on pGEM-T (Promega) and inserted at the *Eco*RI site in a derivative of pGreen II (ver. 0179) that already contained the 35S promoter. This promoter had been inserted as part of a 693 bp *Kpn*I-*Not*I fragment from pGreenII (ver. 0000; http://www.pgreen.ac.uk/). The introduction of plasmid DNA via the process of transformation into cells made competent by treatment with calcium chloride, the isolation of plasmid via alkaline lysis, and the analysis of plasmid using restriction enzyme in combination with agarose gel electrophoresis were done using widely used protocols (Sambrook and Russell 2001). To estimate the yield, plasmid was isolated from 2 OD_600_ units of culture, resuspended in 40 µl of sterile deionized water, and a 2 µl aliquot was analyzed by agarose gel electrophoresis.

### E. coli: growth and measurement of colony forming units (cfu)

*E. coli* DH5α cells containing derivatives of pGreenII (ver. 0179) as described were grown in Luria Bertani broth (Sigma) with kanamycin selection (50 µg/ml) and shaking (200 rpm) at 37°. Cultures of 50 ml were incubated in 250 ml Erlenmeyer flasks, while cultures of 5 ml were grown in 50 ml Falcon conical centrifuge tubes held vertically. Growth was monitored by measuring the optical density of the culture at 600 nm (OD_600_ reading). When the OD_600_ of cultures exceeded 1.0, samples were diluted to ensure that readings were well within the linear range of the spectrophotometer. To determine the number of cfu values, samples of *E. coli* cultures were collected, diluted serially by 10-fold, aliquots spread on the surface of LB agar plates containing kanamycin (50 µg/ml), and incubated overnight. After confirming that the number of colonies on the plates corresponded to the expected 10-fold dilution, the precise number of colonies on a plate with 10–100 colonies was counted and used to determine the number of cfu/ml after correcting for dilution and sample volume spread on plates. To correct for growth, this value was divided by the corresponding OD_600_ value of the culture to give the value of cfu/OD_600_ unit. An OD_600_ unit of 1.0 is the biomass in a 1.0 ml sample with an OD_600_ reading of 1.0. Values of cfu/OD_600_ unit were determined during exponential growth and following overnight culture. Independent measurements were made at least thrice to allow values of average and standard deviation to be calculated. Inoculums to initiate culturing in liquid broth were either cells scraped from individual or multiple colonies derived by the process of transformation, which was started the previous day.

### DNA sequencing: plasmid and chromosome

Plasmids were isolated as described above and sequenced as part of a service provided by Beckman Coulter Genomics (Essex, UK). Chromosomal DNA was isolated as part of a protocol usually used by us to isolate total RNA from *E. coli* (Kime *et al.* 2008). Upon the addition of ethanol to precipitate nucleic acids, chromosomal DNA in the form of a stringy aggregate was removed using a pipette tip, pelleted by brief centrifugation (2 min) in a microfuge, washed with 70% [v/v] ethanol, and dried. It was sequenced to ∼35 × coverage using an Illumina MiSeq as part of a service provided by the Next Generation Sequencing Facility (St James’s University Hospital, Leeds). *E. coli* strain DH10B was used as the reference genome (GenBank: XB000024) and DH5α was sequenced to establish its allelic differences prior to scanning for mutations in the genomes of spontaneous mutants of DH5α using NextGENe software.

### Arabidopsis transformation: confirmation of plasmid transfer

*Arabidopsis* (*Col-0*) was transformed by floral dip (Clough and Bent 1998). 0.6 g of seeds was spread on the surface of MS plates (4.4 g/l Murashige and Skoog plus vitamins; 10 g/l sucrose; 5.5 g/l agar; pH 5.8) containing hygromycin (15 µg/ml) to select transformants (*i.e.*, resistant seedlings), from which DNA was isolated and analyzed by PCR. The sequences of the primer pairs were 5′-GCGTGTCATTGAGAGGTTCG-3’ plus 5′-GTCAAGAGCCTCAAGGAGAG-3’, and 5′-TGCCATGCCCGAAGGTTATG-3’ plus 5′-TGTGTAATCCCAGCAGCAGT-3’. These produced amplicons of 649 bp and 435 bp in the presence of the genes encoding elongation factor 1a and green fluorescent protein, respectively. The *Agrobacterium* culture used for the floral dip (Clough and Bent 1998) was grown at 28° in Luria Bertani (LB) broth (Sigma) containing kanamycin (50 µg/ml), tetracycline (12.5 µg/ml), and gentamycin (40 µg/ml), until an OD_600_ of 1.0 was reached. Cells were pelleted and resuspended in 5% sucrose; 0.05% Silwet-L77 to an OD_600_ of 0.8. *Arabidopsis* plants were grown at 25° under long day conditions for 4 wk and then inverted into the resuspended culture for 1 min. Seeds were harvested and dried.

### Data availability

The vector pViridis and strain MW906 are available upon request from BCCM (http://bccm.belspo.be/). Accession numbers are held by laboratory of P. M. (p.meyer@leeds.ac.uk).

## Results

### Adverse effects of pGreenII on E. coli growth

**Figure 1:**
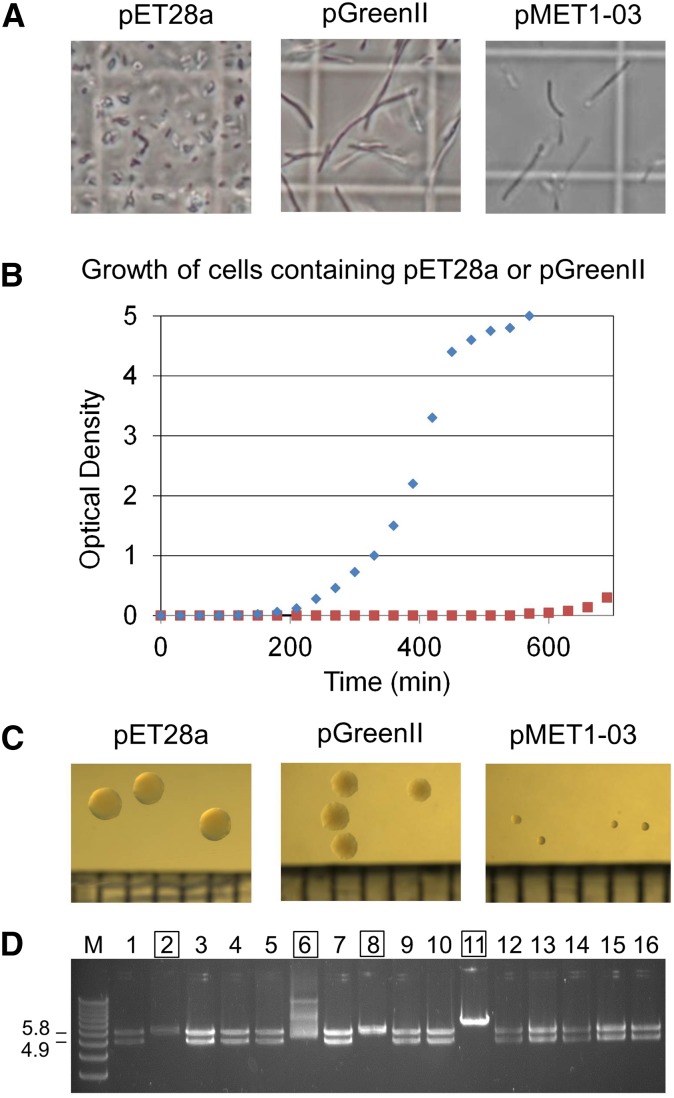
Growth of DH5α cells containing pET28a, pGreenII, and pMET1-03. (A) Cell morphology. Aliquots of culture were viewed using a light microscope under phase contrast at 100 × magnification with an oil immersion objective. A graticule was used to provide a measuring scale. The length of the sides of the visible squares was 50 µm. The length of a typical *E. coli* cell is 2 µm. (B) The growth of *E. coli* using overnight cultures as an inoculum. 50 ml of LB media (containing 50 µg/ml kanamycin) within a 250 ml Erlenmeyer flask was inoculated with 0.2 OD_600_ units of cells from a 5 ml overnight culture (see *Materials and Methods*). Growth was monitored by measuring OD_600_ values. The data-points for pET28a and pGreenII are represented by diamonds and squares, respectively. (C) Colony morphology. The pictures are of primary transformants following overnight incubation. The graduations at the bottom of each image in this panel correspond to 1 mm. (D) Restriction enzyme analysis of a selection of plasmids isolated from mutants that produce large colonies. The lane labeled M contains the 1 kb Plus DNA Ladder (Life Technologies). The mutants are in lanes 1–16. Numbering on the left of the panel indicates the expected sizes of the two fragments produced by *Eco*RI digestion of pMET1-03. The smaller of the two fragments corresponds to the *MET1* cassette destined for plants. Labels that are outlined indicate plasmids with obvious rearrangements. The gel used for electrophoresis was composed of 0.8% [w/v] agarose and stained with ethidium bromide. LB, Luria Bertani; OD, optical density.

The vector pGreenII (version 0179) has an adverse effect on the growth of *E. coli* as evidenced, for example, by extensive filamentation (*i.e.*, incomplete septation) of cells during exponential growth (Figure 1, panel A) and a fourfold increase in the time required for subcultures to reach early-exponential growth (OD_600_ = 0.2) when the inoculum was from a turbid, overnight culture (Figure 1, panel B). The latter suggested a dramatic decrease in cell viability following overnight incubation. The comparator was cells containing pET28a, a vector used as part of one of the most popular systems for the expression of recombinant genes in *E. coli* (Studier and Moffatt 1986; Studier *et al.* 1990). Like pGreenII, pET28a confers kanamycin resistance and has an origin of replication from ColE1.

To quantitate the effects of overnight culture and filamentation, we determined the number of cfu/OD_600_ unit of cells (Table 1). Following overnight incubation in vertical tubes, the cfu value for cells containing pGreenII was three to four orders of magnitude lower than that of cells containing pET28a. This dramatic decrease in the cfu value greatly exceeds the level of filamentation (see below). It is also consistent with poor viability of the overnight culture being the cause of the lengthening of the period it took cells containing pGreenII to reach the early-exponential phase of growth, which coincides with the culture starting to develop noticeable turbidity as judged by eye (Figure 1, panel B). During exponential growth, the average cfu value for cells containing pGreenII was ∼17-fold lower than that for cells containing pET28a, which was consistent with the extent of filamentation observed for cells containing pGreenII (Figure 1, panel A). Despite filamentation, cells containing pGreenII had a doubling time that was only 1.3-fold longer than cells containing pET28a during exponential growth (Table 1).

**Table 1:** Growth parameters of different combinations of strains and plasmids

Combination	Viability Postovernight Culture (cfu/OD_600_)	Colony Forming Units During Exponential Growth (per OD_600_ Unit)	Time (min) to Reach Early Exponential Growth Phase (OD_600_ of 0.2)	Doubling Time During Exponential Growth (min)
DH5α (pET28a)	522E+06 (± 228E+06)	126E+06 (± 41E+06)	220	52
DH5α (pGreenII)	0.08E+06 (± 0.03E+06)	7.6E+06 (± 2.0E+06)	680	67
DH5α (pGreenII-IS5)	77E+06 (± 68E+06)	16.0E+06 (± 2.0E+06)	520	55
DH5α (pViridis)	1,250E+06 (± 351E+06)	105E+06 (± 40E+06)	250	46
MW906 (pGreenII)	5.9E+06 (± 1.1E+06)	84E+06 (± 21E+06)	460	54
BL21(DE3, pGreenII)	0.46E+06	n.d.	n.d.	n.d.

Cfu, colony forming unit; n.d., not determined.

The above experiments were conducted using DH5α, a derivative of *E. coli* K-12 used widely for the purpose of recombining DNA (BRL 1986). However, the effects of pGreenII on growth do not appear to be strain specific. For example, a sharp drop in viability following overnight incubation was observed using BL21 (DE3) (Table 1), a derivate of *E. coli* B used extensively for protein production (Studier *et al.* 1990). The cfu values obtained for DH5α (pET28a) cells during growth were in agreement with those typically reported in the literature (Ausubel 1995). DH5α cells containing pGreenII, in comparison to those containing pET28a, produced slightly smaller colonies on agar plates (Figure 1, panel C), consistent with their longer doubling time and reduced viability after exit from exponential growth (Table 1). The surface and edge of DH5α (pGreenII) colonies also appeared to undulate and be less regular, respectively.

### The selection of spontaneous mutations

The growth defects caused by pGreenII can be compounded when this vector carries an insert. In comparison to DH5α (pGreenII), cells containing pMET1-03, the pGreenII-derived construct that carries *MET1* (for details, see *Materials and Methods*), produced colonies that were noticeably smaller than those of cells containing pGreenII (Figure 1, panel C). More remarkably, it proved impossible to produce reproducibly turbid cultures of cells containing pMET1-03 with overnight incubation. Turbid cultures could be produced when the incubation was extended to 3 d, but when aliquots were spread on agar plates the majority of the resulting colonies were larger (data not shown). This indicated that the combined effects of pGreenII and the *MET1* insert were sufficiently severe to force the selection of spontaneous mutants with improved growth. The analysis of the plasmids from a selection of these mutants revealed that many (4 of 16) had obvious rearrangements involving the *MET1* cassette (4865 bp *Eco*R1 fragment) destined for plants (Figure 1, panel D). Mutants that contain plasmids without obvious changes in restriction fragment length either have chromosome mutations, which we exploited (see below), or plasmids with small indels or nucleotide substitutions. pGreenII without any insert was also able to force the selection of mutants (see below). For this reason, all of the colonies we have shown (Figure 1, panel A) correspond to primary transformants. Moreover, the cultures used for the measurement of the doubling times, and cfu values during exponential growth and following overnight culture, (Table 1) were inoculated using cells obtained from multiple colonies of primary transformants and not overnight cultures.

**Figure 2:**
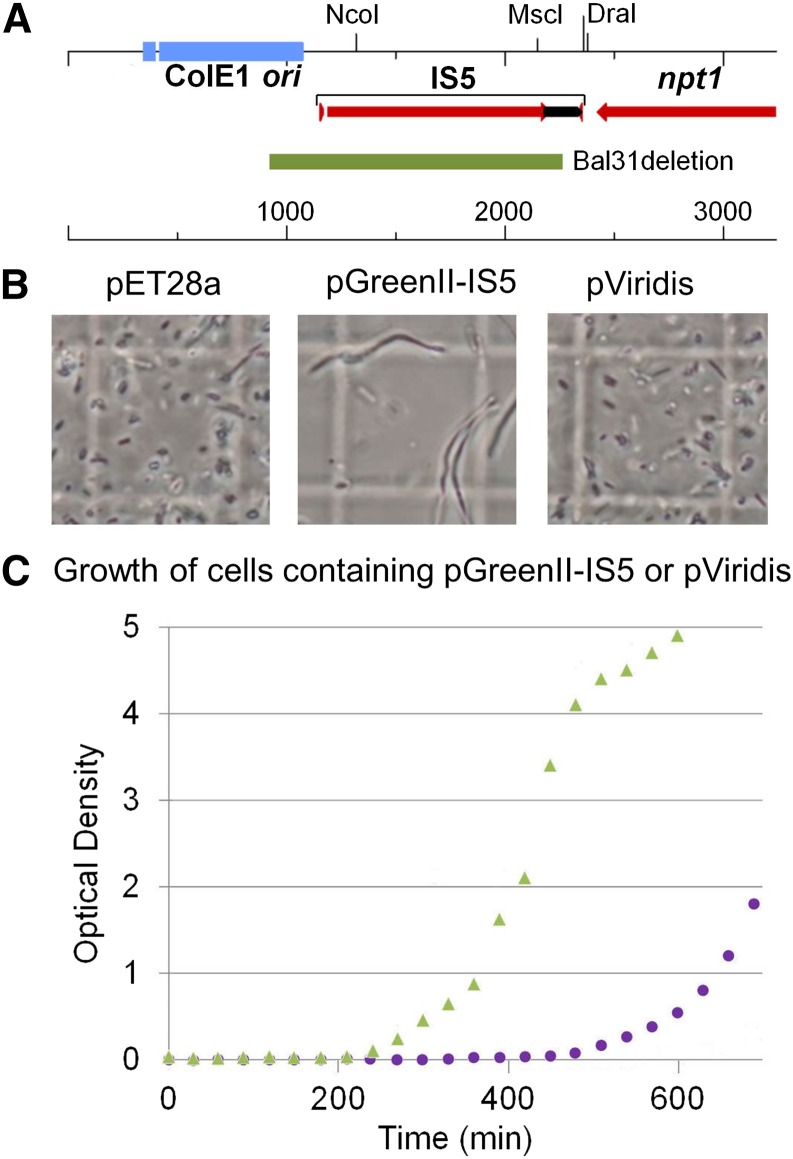
Characterization of plasmids pGreenII-IS5 and pViridis. (A) The region between the ColE1 origin of replication and the 3′ end of *npt1* in pGreenII-IS5. IS5 is integrated 76 bp upstream from the ColE1 *ori* in the middle of the sequence GGATCTTCAC˅ATCCTTTTAA. The horizontal bar labeled “Bal31 deletion” indicates the region in pGreenII-IS5 that is absent in pViridis. The deletion extended for 1308 bp, removed 1130 bp of IS5 retaining only 74 bp of the end downstream of the transposase gene, and extended 103 bp into the ColE1 *ori*. (B) Cell morphology. Imaged as described in Figure 1, panel A. (C) Growth in liquid culture using overnight cultures as an inoculum. The triangles and circles correspond to data-points for cells containing pViridis and pGreenII-IS5, respectively. Compare with the diamonds and squares corresponding to data-points for cells containing pET28a and pGreenII, respectively, in Figure 1.

As a step toward negating the deleterious effects of pGreenII on *E. coli* growth, we selected spontaneous mutants by independently passaging multiple transformants through three cycles of culture using cells from an overnight incubation as the inoculum for the next (for details, see *Materials and Methods*). This led to the isolation of a plasmid that no longer causes such a dramatic drop in cfu values following overnight incubation, even when retransformed into a fresh batch of cells (Table 1). This plasmid was named pGreenII-IS5, as sequencing revealed it had acquired IS5, a 1.2 kbp transposable element (Szybalski 1977; Engler and van Bree 1981), in the region between the ColE1 origin of replication and the stop codon of *npt1* (Figure 2, panel A). This is the location implicated previously in the instability of the original pGreen plasmid (see *Introduction*).

### Complete negation of the growth defects caused by the original pGreenII

Next, to explore the capacity of further changes in the region upstream of the ColE1 origin to reverse the growth defects caused by pGreenII, we introduced deletions using Bal31 into pGreenII-IS5 at the *Nco*I and *Msc*I sites within IS5 and at the two flanking *Dra*I sites (Figure 2, panel A) and then screened > 40 mutants. The cfu values following overnight incubation, as well as the time it took cultures to reach early-exponential growth (using an overnight culture as the inoculum), were determined. In addition to identifying deletions that abolished or left unaffected the beneficial effects of IS5, we identified one that increased the viability of overnight cultures above that provided by the IS5 insertion. Sequencing revealed that the deletion was 1.3 kbp, removed much of the IS5 element, and extended into the ColE1 origin of replication (Figure 2, panel A). The corresponding plasmid was named pViridis.

**Figure 3:**
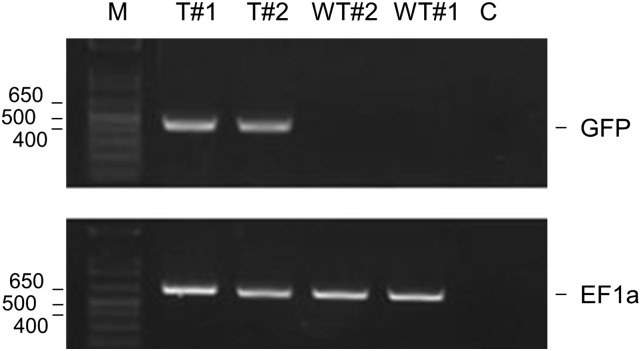
Confirmation of successful *Arabidopsis* transformation. A pViridis-based construct encoding green fluorescent protein (GFP) was introduced by the method of floral dipping. The tissue of two independent transformants (labeled T), *i.e.*, seedlings resistant to hygromycin, were analyzed by PCR for the presence of the gene encoding GFP. An endogenous gene encoding elongation factor 1a (labeled EF1a) and two seedlings of wild-type *Arabidopsis* (labeled WT) were also analyzed by PCR as controls. The sizes of the amplicons corresponding to genes of GFP and EF1a are 435 and 649 bp, respectively. The lane labeled M contains the 1 kb Plus DNA Ladder (Life Technologies), whereas the lane labeled C corresponds to a control reaction to which DNA was not added. PCR, polymerase chain reaction; WT, wild-type.

In comparison to DH5α (pGreenII) cells (Figure 1, panel A), DH5α (pViridis) cells did not produce obvious filaments (Figure 2, panel B) and were straightforward to passage using an overnight culture as the inoculum (Figure 2, panel C). Moreover, their doubling time during exponential growth was 1.50-fold shorter, and the cfu values during exponential growth and following overnight incubation were 14 and 15,600-fold higher, respectively (Table 1). Indeed, the actual values for DH5α (pViridis) cells were very similar to those of DH5α (pET28a) cells (Table 1). Cells containing pGreenII-IS5, the intermediate in the construction of pViridis, produced filaments (Figure 2, panel B) and were still significantly delayed in reaching the exponential phase of growth, although not to the same extent as cells containing pGreenII (Figure 2, panel C and Table 1). The colony morphology of cells containing pViridis was indistinguishable from those of pET28a (data not shown). The pViridis vector has been successfully used by us to clone a number of fragments (M. R. Watson and P. Meyer, unpublished results). Moreover, pViridis constructs have been introduced successfully into Arabidopsis (*Col-0*) (Figure 3).

### A strain that better tolerates pGreenII

As outlined above, we were able to select spontaneous mutations in pGreenII that reduced the deleterious effects of this plasmid on cell growth. During the screening, we also detected chromosomal mutations, *i.e.*, the improved growth characteristics were not linked with the resident plasmid when introduced by transformation into fresh DH5α cells. A strain that better tolerates pGreenII would be beneficial in the propagation and manipulation of the large number of existing constructs based on this vector. Therefore, we repeated the screen (for further details, see *Materials and Methods*), but increased the selection pressure by using cells that carried pMET1-03. The *MET1* insert compounds the effects of pGreenII (Figure 1) by a mechanism that is not dependent on the production of a functional cytosine-DNA-methyltransferase (data not shown). Mutations located in chromosome were identified by showing that improved growth was not transferred with the resident plasmid, and persisted when the strain was cured of the resident plasmid (via culture in the absence of antibiotic) and retransformed with a fresh batch of pGreenII.

**Figure 4:**
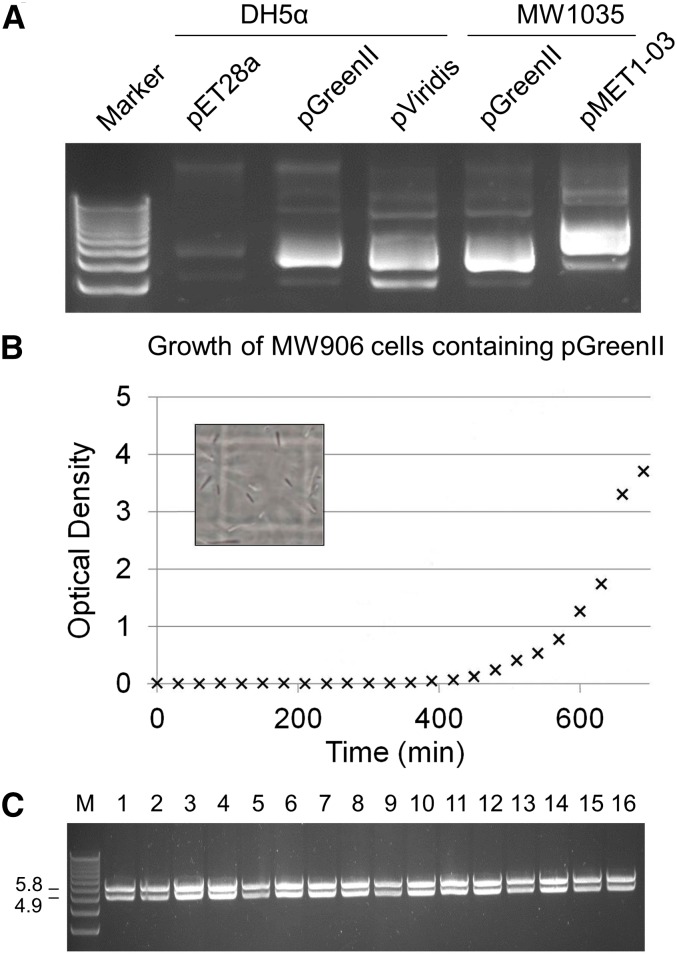
Characterization of strain MW906. (A) Plasmid yield. Labeling at the top of the panel indicates the combinations of strains and plasmids. For each combination, plasmid DNA was isolated from 2 OD_600_ units of an overnight culture using a standard protocol (for details, see *Material and Methods*). The marker was the 1 kb Plus DNA Ladder (Life Technologies). The gel used for electrophoresis was composed of 0.8% [w/v] agarose and stained with ethidium bromide. (B) Growth in liquid culture using overnight cultures as an inoculum. The crosses correspond to data-points for MW906 cells containing pGreenII. Compare with the diamonds and squares corresponding to data-points for DH5α cells containing pET28a (Figure 1) and pGreenII (Figure 3), respectively. The inset shows the morphology of MW906 (pGreenII) cells, as imaged in Figure 1, panel A. (C) Restriction enzyme analysis of plasmid from a selection of clones isolated from a culture of MW906 (pMET1-03) following overnight incubation (labeled 1–16). The lane labeled M contains the 1 kb Plus DNA Ladder (Life Technologies). Numbering on the left of the panel indicates the expected sizes of the two fragments produced by *Eco*RI digestion of pMET1-03. The smaller of the two fragments corresponds to the *MET1* cassette destined for plants. The gel used for electrophoresis was composed of 0.8% [w/v] agarose and stained with ethidium bromide.

Next, we assayed chromosomal mutants for plasmid yield to avoid mutations that alleviated the deleterious effects of pGreenII by reducing its copy number. This revealed a mutant strain, now designated MW906, which yielded an amount of pGreenII at least comparable to that obtained from DH5α (Figure 4, panel A). Included in this analysis were DH5α cells containing pET28a and pViridis, and MW906 cells containing pGreenII and pMET1-03. Sequencing of MW906 located the spontaneous mutation to the *pcnB* gene (Liu and Parkinson 1989), which encodes an RNA poly(A) polymerase (Cao and Sarkar 1992). The mutation caused a glycine to serine substitution at position 67 (*i.e.*, a G67S mutation). In comparison to the equivalent data for DH5α (pMET1-03) cells (Figure 1), MW906 (pMET1-03) cells took considerably less time to reach early-exponential growth using an overnight culture as the inoculum (Figure 4, panel B) and did not appear to produce filaments, at least to the same extent (see inset, Figure 4, panel B) The latter interpretation is consistent with the finding that the cfu values during exponential growth are higher (Table 1). The doubling time during exponential growth and viability following overnight incubation were also improved (Table 1). More importantly, with regard to maintaining the integrity of DNA intended for plants, turbid cultures of MW906 (pMET1-03) cells could be produced readily by overnight incubation, and so far the analysis of plasmids from clones isolated at the end of overnight incubation has failed to identify any rearrangement of the plasmid (for examples, see Figure 4, panel C).

## Discussion

The plasmid vector pGreenII, which is widely used in the production of stable plant transformants, is shown herein to predispose constructs to the acquisition of mutations (Figure 1) despite its earlier revision (Hellens and Mullineaux 2000). This predisposition arises from pGreenII having an adverse effect on the growth of *E. coli*. It perturbs normal cell division resulting in the production of long filaments (Figure 1), a phenomenon associated with stressed cells (Justice *et al.* 2008), and causes a dramatic reduction in cell viability following overnight incubation (Table 1). This is far from ideal as the insertion of DNA into plasmids can itself affect the growth of *E. coli* through increased metabolic burden and the acquisition of activities that perturb cellular functions (Bentley *et al.* 2009; Rosano and Ceccarelli 2014). In our case, the insertion of a 4605 bp fragment containing the cDNA of the plant DNA methyltransferase 1 (MET1) into pGreenII generated sufficient selective pressure for spontaneous, faster-growing mutants to dominate cultures, such as those used to isolate plasmid DNA by mini preparation, when with extended incubation cultures eventually became turbid (Figure 1).

Given the clear need to avoid a selective pressure that can affect the integrity of DNA destined for plants, we successfully isolated and characterized a derivative of pGreenII that no longer produces any of the growth effects described above. Cells containing this plasmid, which we have called pViridis, grow as well as cells containing pET28a (Figure 2), a plasmid used widely for protein production in *E. coli* (Studier and Moffatt 1986; Studier *et al.* 1990). The doubling times, cell morphology, and viability after overnight incubation for cells carrying pViridis or pET28a are indistinguishable (Figure 2 and Table 1). Moreover, we have been able to clone a number of DNA fragments into pViridis (M. R. Watson and P. Meyer, unpublished results). The yield of pViridis is comparable to that of pGreenII using a standard protocol for the isolation of plasmid DNA (Figure 4).

While the new pViridis vector represents a valuable tool for the plant scientific community, many labs have already produced constructs using the highly popular pGreenII vector, most likely not being aware of its susceptibility to cause mutation, which of course impacts the long-term conservation and fidelity of pGreenII-based constructs. Therefore, we successfully selected MW906, a DH5α-derived strain, which better tolerates pGreenII-based constructs without causing a reduction in plasmid yield. DH5α was chosen as the background strain for the selection as it is commonly used for DNA cloning in *E. coli* (BRL 1986). The viability of MW906 (*cf*. DH5α) cells containing pGreenII increased by two orders of magnitude, while the filamentous phenotype was reduced, although not completely eliminated. The superiority of strain MW906 was also demonstrated by the relative ease with which it can be propagated when carrying pMET1-03 and the greater stability of pMET1-03. With regard to the latter, we failed to identify any isolates containing plasmid with obvious rearrangements at the end of batch culture (Figure 4). However, given the choice of using the combination of MW906 and pGreenII or DH5α and pViridis for the construction of new cassettes for delivery into plants, we would suggest the latter combination. This is because MW906 (pGreenII) does not grow as well as DH5α (pViridis); consequently, the selective pressure for mutation has not been completely removed using MW906 (pGreenII). However, it should be noted that while the G67S mutation does not affect the yield of constructs based on pGreenII, it does reduce the yield of constructs based on pViridis (data not shown). Thus, we recommend always using DH5α for the propagation of constructs based on pViridis. Other strains that are wild-type with regard to the *pcnB* gene might also be suitable.

At the present time, we can only speculate on the cause of the growth defects conferred by pGreenII. However, as part of our investigation, we found that the spontaneous insertion of IS5, a 1.2 kbp transposable element, in the region upstream of the ColE1 origin of replication (and downstream of the *npt1* gene, which confers kanamycin resistance) increased the viability of cells following overnight culture by around three orders of magnitude. The cfu values for DH5α (pGreenII-IS5) were lower than those for pET28a, but no more than could be explained by filamentation, which was not suppressed. The location of IS5 suggests that some aspect of the replication of pGreenII is abnormal. This is supported by the fact that the deletion which produced pViridis extended 103 bp into the ColE1 origin of replication, removing the endogenous promoter and 5′ end of the RNAII, which is the primer of plasmid replication (Polisky 1988). In addition, the chromosomal mutation that permits better tolerance of pGreenII-based constructs causes an amino acid substitution within *pcnB* (Lopilato *et al.* 1986), which controls plasmid copy number by promoting the degradation of RNAI (Xu *et al.* 1993; He *et al.* 1993), the antisense RNA regulator of RNAII activity (Cesareni *et al.* 1991). The genes encoding RNAI and RNAII overlap; thus, the deletion in pViridis causes truncation of 45 nucleotides from the 3′ end of RNAI, as well as the 5′ end of RNAII. The deletion also illustrates the functional plasticity of the RNA components of the ColE1 replication machinery. We have found that other mutations in *pcnB* can alleviate the growth defects conferred by pGreenII, but not without reducing plasmid yield (data not shown). Thus, the G67S mutation in MW1053 appears to be unique in correcting a defect in the replication of pGreenII, while managing to provide a normal balance between the activities of RNAI and RNAII. Consistent with this notion, when the proposed imbalance is corrected in the case of pViridis, the effect of the *pcnB* mutation in MW906, instead of establishing balanced regulation, causes a reduction in plasmid copy number (data not shown).

The source of the original instability reported for pGreen might also have been related to plasmid replication (Hellens and Mullineaux 2000). It was reported that several ’enlarged’ plasmids had *E. coli* chromosomal DNA inserted in the ColE1 *ori*, and reasoned that the selective pressure may have been the inefficient termination of transcription from the convergent *npt1* gene (Figure 2). As indicated previously, the region between the ColE1 origin and the stop codon of the *npt1* gene in pGreen was replaced with the corresponding sequence from pBluescript to produce pGreenII. However, we can report that the insertion of a strong transcriptional terminator (BBa_B0010, iGEM Registry of Standard Biological Parts; Campos 2012) between the ColE1 *ori* and the 3′ end of the *npt1* gene in pGreenII had no discernible effect on the ability of pGreenII to cause growth defects (data not shown). Indeed, in pViridis, transcription from *npt1* may contribute to the transcription of RNAII in the absence of the promoter that normally produces the primer of replication.
